# Effects of Pregnancy and Lactation on Iron Metabolism in Rats

**DOI:** 10.1155/2015/105325

**Published:** 2015-12-16

**Authors:** Guofen Gao, Shang-Yuan Liu, Hui-Jie Wang, Tian-Wei Zhang, Peng Yu, Xiang-Lin Duan, Shu-E Zhao, Yan-Zhong Chang

**Affiliations:** ^1^Laboratory of Molecular Iron Metabolism, College of Life Science, Hebei Normal University, Shijiazhuang, Hebei 050024, China; ^2^The 3rd Hospital of Hebei Medical University, Shijiazhuang, Hebei 050017, China

## Abstract

In female, inadequate iron supply is a highly prevalent problem that often leads to iron-deficiency anemia. This study aimed to understand the effects of pregnancy and lactation on iron metabolism. Rats with different days of gestation and lactation were used to determine the variations in iron stores and serum iron level and the changes in expression of iron metabolism-related proteins, including ferritin, ferroportin 1 (FPN1), ceruloplasmin (Cp), divalent metal transporter 1 (DMT1), transferrin receptor 1 (TfR1), and the major iron-regulatory molecule—hepcidin. We found that iron stores decline dramatically at late-pregnancy period, and the low iron store status persists throughout the lactation period. The significantly increased FPN1 level in small intestine facilitates digestive iron absorption, which maintains the serum iron concentration at a near-normal level to meet the increase of iron requirements. Moreover, a significant decrease of hepcidin expression is observed during late-pregnancy and early-lactation stages, suggesting the important regulatory role that hepcidin plays in iron metabolism during pregnancy and lactation. These results are fundamental to the understanding of iron homeostasis during pregnancy and lactation and may provide experimental bases for future studies to identify key molecules expressed during these special periods that regulate the expression of hepcidin, to eventually improve the iron-deficiency status.

## 1. Introduction

Iron is an essential trace element for almost all life forms on earth. In human, iron and iron-containing compounds play critical roles in several biologically important processes, including oxygen transport and storage, electron transport, energy metabolism, and antioxidant and DNA synthesis. Iron deficiency is the most common nutrient deficiency of human in the world, which, at its worst-case scenario, could result in iron-deficiency anemia due to inadequate iron supply for normal red blood cell formation [1, 2]. It has been found that iron demand increases notably in pregnant women because of the expansion of the maternal erythrocyte mass and the growth and development of the fetus [2, 3]. The increased iron demand is met initially through the use of maternal iron stores primarily from the liver [4]. However, as iron stores are depleted, if functional iron absorbed from the diet could not maintain an adequate iron supply for both the mother and the fetus [3], iron-deficiency anemia would occur [2]. Although dysregulations of iron metabolism in pregnant women were described as early as the 19th century [2, 3, 5], few studies were conducted ever since at the molecular level to investigate the molecules involved in the regulation of iron homeostasis.

The function of an iron-regulatory hormone, hepcidin, a 25-amino acid peptide produced by liver, has been extensively studied since its discovery in 2001 [6, 7]. Hepcidin binds to the cellular iron exporter ferroportin 1 (FPN1), results in its internalization and degradation [8, 9], thereby inhibits intestinal iron absorption from enterocytes, and reduces iron release from macrophages [10]. When the iron level in the body is raised, the hepcidin expression is upregulated [11], which subsequently leads to the decrease of iron absorption and iron release from macrophages, and reduces iron in the circulation [12]. To the contrary, when the iron level in the body is low, the hepcidin expression is downregulated [13]. Studies on transgenic mice have showed that hepcidin downregulation leads to iron overload [14, 15], and overexpression of hepcidin leads to severe iron deficiency and anemia [16], indicating hepcidin's function on maintaining iron homeostasis [17, 18]. In 2004, a study by Millard et al. showed that the increased iron absorption during pregnancy in rats was associated with decreased hepcidin expression [5]. However, no comprehensive studies were done on the function of hepcidin in the regulation of iron metabolism at various stages of pregnancy. Furthermore, none of the reports was seen regarding the iron status of the postpartum females during their lactation period, nor were the changes in expression of iron metabolism-related proteins and the role of hepcidin in it reported. Therefore, in this study, we investigated the changes of hepatic iron content at various stages of pregnancy and lactation in rats, the involvement of iron transport and efflux proteins, and the potential role of hepcidin regulation in iron metabolism. These studies may provide experimental and theoretical bases for a more comprehensive understanding of abnormal iron metabolism during pregnancy and lactation to prevent the iron-deficiency status for women during the pregnancy and lactation.

## 2. Methods

### 2.1. Animals

Female Sprague-Dawley (SD) rats were obtained from the Hebei Medical University Animal Breeding Center (Hebei, China), fed with a standard rodent pellet diet (370 mg iron/kg), and time-mated at 10–12 weeks of age. Number of experimental animals' certificate of conformity is 604170. All experiments were approved by the Animal Care and Use Committee of Hebei Science and Technical Bureau, China, and by the Animal Ethics Committee of Hebei Normal University. Rats were housed at ambient temperature of 22°C–24°C with relative humidity of 45%–55% and free access to water. Rats were sacrificed at 9, 15, 18, and 21 days of gestation or 1, 7, 14, and 21 days of lactation. Nonpregnant female rats were used as controls. 12 female SD rats in each stage of gestation or lactation were used for analysis. Rats were anesthetized (0.04% sodium pentobarbital, 1 mL/100 g) and samples of liver and duodenum were dissected and snap-frozen in liquid nitrogen.

### 2.2. Serum Iron and Tissue Non-Heme Iron Measurements

Blood samples were collected from rat tails and centrifuged at 845 g for 20 min at 4°C, and then the supernatant was collected and serum was obtained [20]. Serum iron (SI) levels, unsaturated iron-binding capacity (UIBC), total iron-binding capacity (TIBC), and transferrin saturation (TS) of the serum were assayed according to the manufacturer's instructions of the iron and iron-binding capacity reagent kit (Nanjing Jiancheng, Nanjing, China). The absorbance was read at 520 nm by using a 722S visible spectrophotometer (Shanghai Lengguang, Shanghai, China). For the tissue non-heme iron determination, liver and spleen samples were dried overnight at 110°C and extracted with acid. Non-heme iron content was determined using a colorimetric assay as described previously [19].

### 2.3. Western Blot Analysis

Tissues were washed and homogenized in RIPA buffer (50 mM Tris-HCl, pH 8.0, 150 mM NaCl, 5 mM EDTA, and 1% NP-40) including protease inhibitors (1 mM PMSF, 10 *μ*g/mL leupeptin, 10 mg/mL pepstatin A, and 1 mg/mL antipain) and phosphatase inhibitors (10 *μ*L/mL phosphatase inhibitor cocktails; Biomed, Beijing, China). Protein concentration was determined using the BCA Protein Assay Kit (Vigorous, Beijing, China). Protein extracts (35 *μ*g) were diluted in Laemmli buffer (Sigma-Aldrich, St. Louis, USA), incubated for 5 min at 95°C, and subjected to 10% SDS-PAGE. Protein bands were transferred to nitrocellulose membranes (Bio-Rad, Hercules, CA, USA). The membranes were blocked in 5% nonfat milk in TBS-T buffer (20 mM Tris-HCl, pH 7.6, 137 mM NaCl, and 0.1% Tween-20) for 2 hours at room temperature and incubated with primary antibody overnight at 4°C. After washing with TBS-T for three times, the membranes were incubated for 2 hours at room temperature in the secondary antibody conjugated to horseradish peroxide (ZhongShan Biotechnology, Beijing, China). Enzyme activity was visualized by an enhanced chemiluminescence method (Pierce Biotechnology, Rockford, IL, USA), and quantification of proteins was done by normalizing the intensity of the specific probe band to *β*-actin using Quantity One software (Multi Gauge V3.1, Fujifilm Life Science, Japan). TfR1 antibody was purchased from Invitrogen (Shanghai, China). L-ferritin antibody, DMT1(+IRE) and DMT1(−IRE) antibodies, and anti-FPN1, anti-Cp, and TfR2 antibodies were purchased from Alpha Diagnostic International (San Antonio, TX, USA). *β*-actin antibody was obtained from Sigma-Aldrich (Saint Louis, MO, USA).

### 2.4. Quantitative Real-Time PCR

Liver samples were dissected for RNA isolation, rapidly frozen, and stored in liquid nitrogen. Total RNA was extracted and purified using TRIzol Reagent (Invitrogen, CA, USA). The relative purity of isolated total RNA was assessed spectrophotometrically to make sure the *A*260/*A*280 nm ratio exceeded 1.9 for all preparations. Total RNA was reverse-transcribed to cDNA using a reverse transcription kit (Takara, Dalian, China) with oligo(dT) primers according to the manufacturer's instructions. The resulting cDNA samples were analyzed by quantitative real-time PCR using SYBR green as the fluorescence dye according to the manufacturer's instructions (GenStar Biosolutions, Beijing, China). Relative quantities of target genes were normalized to the respective *β*-actin level in each sample. The primer sequences were as follows [21]:

 hepcidin forward primer 5′-CAAGATGGCACTAAGCACTCG-3′;
 hepcidin reverse primer 5′-GCTGGGGTAGGACAGGAATAA-3′;
 
*β*-actin forward primer 5′-GGTCACCCACACTGTGCCCATCTA-3′;
 
*β*-actin reverse primer 5′-GACCGTCAGGCAGCTCACATAGCTCT-3′.

### 2.5. Statistical Analysis

All statistical analyses were completed using SPSS 21.0 software. Results are presented as mean ± SD. The statistical analyses of group differences were assessed by a one-way analysis of variance (ANOVA) followed by* post hoc* Tukey tests. *P* values of <0.05 were considered statistically significant.

## 3. Results

### 3.1. Iron Stores Significantly Decreased at Late-Pregnancy and Early-Lactation Stages

**Figure 1:**
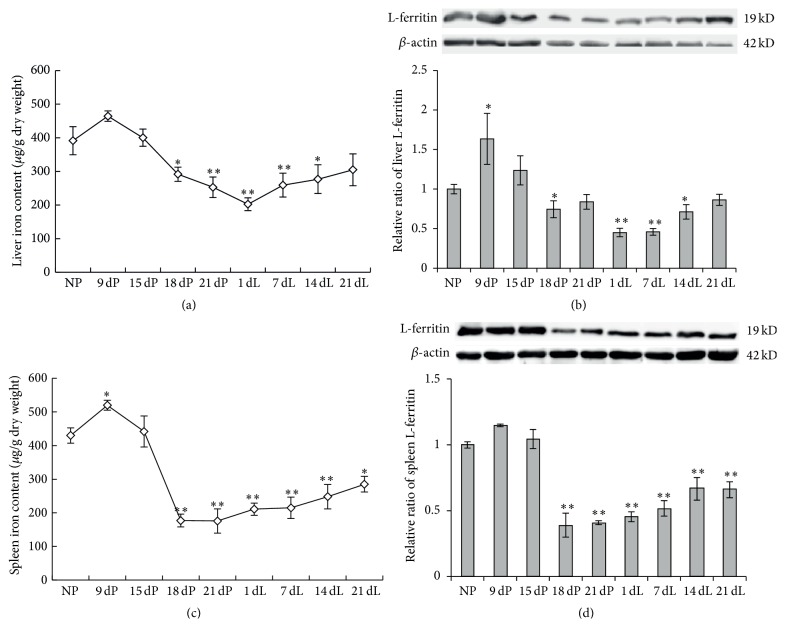
The levels of non-heme iron and L-ferritin in liver and spleen of SD rats at various stages of pregnancy and lactation. Rats were sacrificed at 9, 15, 18, and 21 days of pregnancy (9 dP to 21 dP) or 1, 7, 14, and 21 days of lactation (1 dL to 21 dL). Nonpregnant female rats were used as control. Non-heme iron content in liver (a) and spleen (b) was determined using a colorimetric assay [19]; L-ferritin expression level in liver (c) and spleen (d) was determined by Western blot. A representative blot image for each protein and its respective *β*-actin was shown. The expression levels in different groups were calculated by normalizing the specific bands to the respective *β*-actin bands. The relative expression levels as compared to NP control group were calculated and expressed as mean ± SD. ^*∗∗*^ *P* < 0.01 versus NP; ^*∗*^ *P* < 0.05 versus NP. *n* = 12.

To confirm the reported iron-deficient status in females during pregnancy and to determine the iron level during lactation, we measured the iron stores and serum iron status of rats at different stages of gestation and lactation. We found that the hepatic non-heme iron level seemed increased slightly at early-pregnancy stage as shown in the 9-day pregnancy (9 dP) group (Figure 1(a)). Iron levels decreased significantly after that and reached the lowest level in the 1-day lactation (1 dL) group, which was approximately 51.8% as compared to the nonpregnancy (NP) control rats (*P* < 0.01). During the lactation period, we found a progressive increase in iron levels (Figure 1(a), 1 dL to 21 dL), which were still well below the control level even in the 14-day lactation group (*P* < 0.05). The expression level of ferritin, the primary intracellular iron-storage protein, reflected the level of iron stores, as the L-ferritin level was found to be consistent with the changes of the iron level (Figure 1(b)). More specifically, the L-ferritin was expressed to the highest level in the 9 dP group, declined to the lowest level in the group of 1 dL, and increased slowly over the lactation period and returned to the near-normal level in the 21 dL group (Figure 1(b)).

The splenic non-heme iron levels showed similar changing patterns to hepatic iron, increasing at early-pregnancy and declining significantly at late-pregnancy and lactating stages (Figure 1(c)). The lowest level of iron stores appeared at 21 dP, which is only about 40% of the nonpregnant controls. The expression level of L-ferritin, as shown in Figure 1(d), further confirmed the observed iron levels in spleen during different stages of pregnancy and lactation. All these results indicated that iron stores in both the liver and the spleen of rats increased slightly at beginning of pregnancy but decreased significantly at late-pregnancy stage, and then the low storage iron levels persisted throughout the lactation period. The changes of iron stores in liver and spleen may imply that more iron was released from iron stores and transported to the blood at late-pregnancy and early-lactation stages to meet the high iron demand of the growing fetus.

### 3.2. Serum Iron Status Did Not Alter during Pregnancy and Lactation

When we determine the serum iron status of these rats, we surprisingly found that neither the pregnant nor the lactating rats showed any decrease in the serum iron concentration (Table 1). The serum iron (SI) level showed a small increase in the 9 dP rats, correlating with the increase of hepatic and splenic iron stores at 9 dP. After 9 dP, in contrast to the low level of iron stores, the serum iron concentrations remained at the near-normal level, which may increase slightly but were not statistically significant. The unsaturated iron-binding capacity (UIBC) and total iron-binding capacity (TIBC) of the sera did not show obvious differences between the NP controls and the various pregnancy and lactation groups (Table 1). The transferrin saturation (TS) of the sera seemed increased for the pregnancy and lactation groups, but none of them had statistically significant changes. From this observation, we hypothesized that the ample amount of serum iron may due to the release of iron stores in liver and spleen and/or the absorption of dietary iron by the intestine.

### 3.3. Increased Actions of Iron Transport Proteins in Liver and Spleen Are Observed

**Figure 2:**
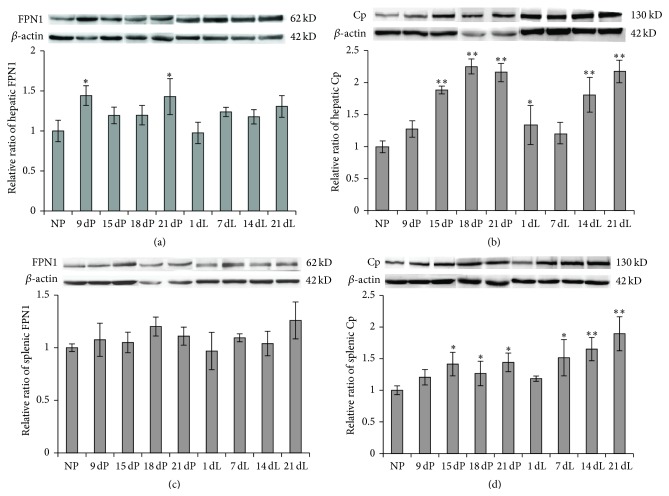
Protein expression levels of FPN1 and Cp in liver (a and b) and spleen (c and d) of SD rats at various stages of pregnancy and lactation. The relative expression levels determined by Western blot were normalized to the respective *β*-actin levels, and their ratios as compared to NP control group were calculated and expressed as mean ± SD. ^*∗∗*^ *P* < 0.01 versus NP; ^*∗*^ *P* < 0.05 versus NP. *n* = 12.

Since the tissue iron level is tightly associated with the expression levels of iron transport proteins, we determined the expression of iron release and intake related proteins at various pregnancy and lactation stages. First we checked the levels of iron release proteins ferroportin 1 (FPN1) and ceruloplasmin (Cp) in liver. We found that the FPN1 level increased in all groups of pregnant and lactating rats, but only the 9 dP and 21 dP groups showed statistically significant increase, approximately 44% higher than the control group (Figure 2(a)), whereas the Cp level increased extremely significantly in the late-pregnancy (15 dP to 21 dP) and lactation stages, except the 7 dL group (Figure 2(b)). The highest level was at 18 dP, which reached >2-times that of the control (Figure 2(b)). When comparing the changes of Cp and FPN1 levels, it seemed that the change of Cp level was more significant. The same change patterns of FPN1 and Cp were also observed in spleen (Figures 2(c) and 2(d)). Since the iron stores were significantly decreased at late-pregnancy and early-lactation periods, higher iron releases from liver and spleen were expected to be observed. Thus, the weak change of FPN1 and strong change of Cp expression may suggest that the catalytic function of Cp stabilized the iron exporter FPN1, and the longer retention time of FPN1 on the cell surface increased cellular iron release subsequently [22].

**Figure 3:**
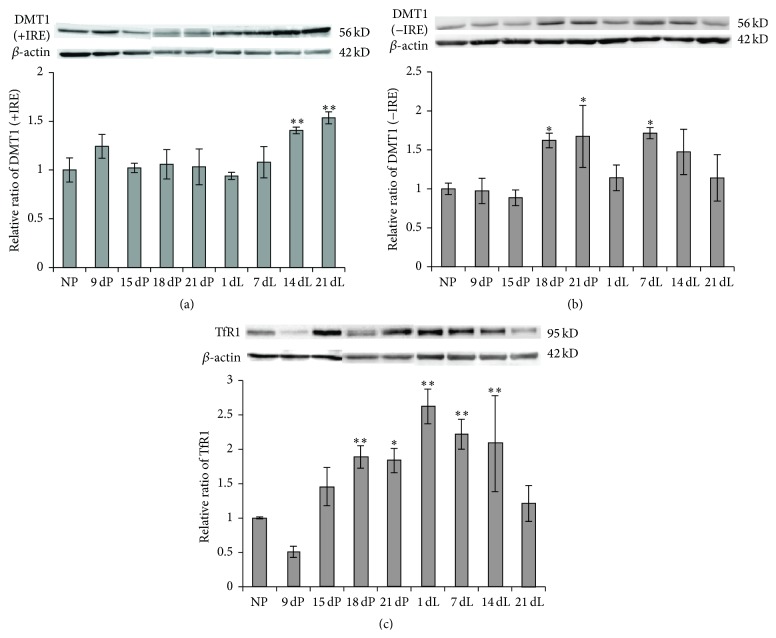
The DMT1(+IRE), DMT1(−IRE), and TfR1 protein levels in liver of SD rats at various stages of pregnancy and lactation. Protein expression level was determined by Western blot. The relative expression levels were normalized to the respective *β*-actin levels, and their ratios as compared to NP control group were calculated and expressed as mean ± SD. ^*∗∗*^ *P* < 0.01 versus NP; ^*∗*^ *P* < 0.05 versus NP. *n* = 12.

We then checked the level of iron intake protein divalent metal transporter 1 (DMT1) with (+) and without (−) the iron responsive element (IRE) in its 3′ untranslated region (UTR) of the transcribed mRNA. The DMT1(+IRE) level increased notably only at the late-lactation stages (14 dL and 21 dL groups), and changes in the other groups were not significant (Figure 3(a)). Meanwhile DMT1(−IRE) levels increased significantly in the 18 dP, 21 dP, and 7 dL groups (Figure 3(b)), which seemed to compensate the relatively low level of DMT1(+IRE). The total DMT1 level showed an increase during pregnancy and lactation overall. We also determined the level of another important iron intake molecule, transferrin receptor 1 (TfR1). As shown in Figure 3(c), TfR1 increased substantially during late-pregnancy and early-lactation (18 dP to 14 dL) stages. The highest level was shown in 1 dL group, which was ~2.6-times that of the control group. From 1 dL to 21 dL, the expression of TfR1 gradually reduced and dropped to the normal level in the 21 dL group. The increase of these iron intake proteins suggests that iron metabolism in liver is more active during pregnancy and lactation.

### 3.4. FPN1 Expression in Small Intestine Substantially Increased to Facilitate Iron Absorption

**Figure 4:**
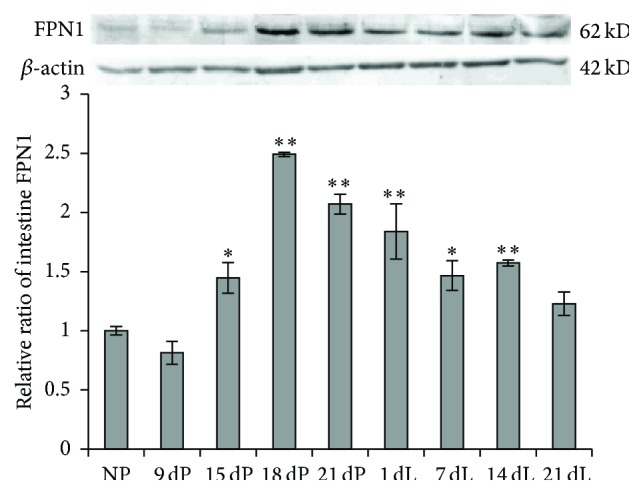
The FPN1 protein levels in small intestine of SD rats at various stages of pregnancy and lactation. Protein expression level was determined by Western blot. The expression levels in different groups were normalized to the respective *β*-actin levels, and the relative expression levels as compared to NP control group were calculated and expressed as mean ± SD. ^*∗∗*^ *P* < 0.01 versus NP; ^*∗*^ *P* < 0.05 versus NP. *n* = 12.

During pregnancy and lactation, the high iron demand of the growing fetus and offspring is acquired from maternal sources. Since the serum iron levels of the pregnant and lactating rats did not reduce, we hypothesized that small intestine would absorb more iron to meet the need of the maternal body. Therefore we examined the expression level of the only known iron efflux protein FPN1 in small intestine. We found that FPN1 levels elevated significantly at late-pregnancy and early-lactation periods (Figure 4). From 15 dP, it increased markedly and reached the highest level in the 18 dP group, which is about 2.5-times that of the NP group. From 21 dP to 21 dL, FPN1 levels decreased gradually but were still significantly higher than the NP group till 14 dL (*P* < 0.01). This indicated that more iron is released from the intestinal epithelial cells to the circulation to meet the high iron demand of the body during pregnancy and lactation.

### 3.5. Hepcidin mRNA Level Significantly Declines at Late-Pregnancy and Early-Lactation Stages

**Figure 5:**
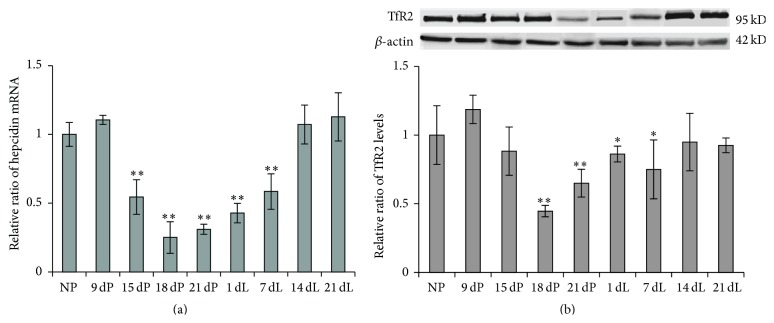
The hepcidin mRNA and TfR2 levels in liver of SD rats at various stages of pregnancy and lactation. The mRNA level was determined by quantitative real-time PCR (a). Protein levels determined by Western blot (b). Their levels in different groups were normalized to the respective *β*-actin levels, and the relative mRNA levels as compared to NP control were calculated and expressed as mean ± SD (b). ^*∗∗*^ *P* < 0.01 versus NP; ^*∗*^ *P* < 0.05 versus NP. *n* = 12.

Since intestinal FPN1 level is primarily regulated by the iron-regulatory molecule—hepcidin, which is mainly produced in liver, we measured the hepatic hepcidin mRNA level. We found that, as predicted, the change trend of hepcidin mRNA (Figure 5(a)) is exactly the opposite of intestinal FPN1 (Figure 4) at almost every corresponding stage, attesting its regulatory role to FPN1 expression. The hepcidin mRNA level declined significantly at late-pregnancy and early-lactation stages (15 dP to 7 dL). It reached the lowest level in the 18 dP group, which was 80% lower than the control group. From 18 dP to 21 dL, hepcidin gradually increased and returned to the normal level in the 21 dL group. The reductions of hepcidin in the 15 dP, 18 dP, 21 dP, 1 dL, and 7 dL groups were extremely significant (*P* < 0.01), while no significances were shown in the 14 dL and 21 dL groups. When comparing the level of hepatic hepcidin to hepatic iron content (Figures 5(a) and 1(a)), we found that the alterations of the two correlated very well at each time point, suggesting that the expression of hepcidin mainly senses and responds to the change of iron store status [23, 24], but not the iron concentration in the blood.

The precise mechanisms for the “iron sensing” role of the liver that control hepcidin production remain to be elucidated; however, studies have reported several iron sensing proteins in liver, including hemochromatosis protein (HFE), HJV, TfR2, and IRP [25]. Here, we determined the expression level of liver TfR2. TfR2 is considered as an upstream signal molecule, possibly in conjugation with HFE, to active hepcidin expression [26, 27]. As shown in Figure 5(b), the TfR2 level began to decline from 9 dP and reached the lowest level in 18 dP group, which was ~55.4% lower than the control group. From 18 dP to 7 dL, the TfR2 levels were still well below the nonpregnant level (*P* < 0.05). TfR2 gradually increased and returned to the near-normal level at 21 dL. This reduction in TfR2 was correlated with the reduction of hepcidin, suggesting the reduced level of TfR2 might be one of the causative factors for the downregulation of hepcidin expression.

## 4. Discussion

Iron deficiency is one of the most prevalent nutrient deficiency problems among pregnant women [28]. Our studies in rats validated this phenomenon. The iron levels in both liver and spleen of pregnant rats showed a small increase at the early stage of pregnancy and decreased dramatically in late-pregnancy stage (Figures 1(a) and 1(c)). The liver began to recover from the iron challenges after birth as shown by the gradually elevated iron level over the lactation period, but iron levels were still significantly below the progestation level (Figures 1(a) and 1(c)). This suggests that the rats had adequate iron to support iron need of the growing fetus until approximately half-way through pregnancy. After this, however, the fetal demands are too high [29], and the rat continues to supply the fetus at the expense of reducing iron stores in the liver and spleen. Due to the high iron needs of the growing fetus during late-pregnancy stage, as well as the iron transfer in the form of lactoferrin during lactation, the insufficient iron stores not only appear during pregnancy, but also persist universally through lactation period. However, the serum iron concentration and transferrin saturation of the rats did not show any decrease over both pregnancy and lactation periods (Table 1). This further inferred that iron was transported to the fetus during pregnancy, and the transport continues during lactation to supply nutrients for the offspring.

The markedly increased intestinal FPN1 level (Figure 4) during the late-pregnancy and lactation periods indicates an increase in iron efflux from enterocytes to blood, which increases iron absorption from the small intestine, and is supposed to be a feedback of the low iron level during pregnancy and lactation to compensate the high iron demand and to prevent the depletion of iron stores [3, 4]. Besides, we found that both iron intake and iron release proteins in liver had varying degrees of changes over pregnancy and lactation (Figures 2 and 3). An overall upregulatory tendency was shown in all iron transport proteins, indicating an active iron transport status in liver to compensate the high iron demand of the maternal body. The upregulation of DMT1 and TfR1 increased cellular iron intake to prevent the depletion of iron stores in the body (Figure 3), while the elevated cellular iron release from iron stores, possibly through FPN1, may compensate the circulating iron level (Figure 2). In our observation, the increase of FPN1 was rather minor as compared to the increase of Cp in both liver and spleen during late-pregnancy and lactation stages (Figure 2). As the iron stores indeed substantially decreased and the FPN1 is the only known iron efflux protein, it may imply that it was the catalytic function of Cp that stabilized FPN1 on the cell surface [22]; thereby the longer retention time of FPN1 increased cellular iron release subsequently.

As is known, hepcidin, a circulating regulatory hormone peptide produced mainly by hepatocytes, functions as the master regulator of cellular iron efflux by controlling the amount of FPN1 [5, 30]. It has been reported that expression of both serum hepcidin and liver hepcidin is lower during pregnancy and postpartum [31], presumably to ensure greater iron bioavailability to the mother and fetus. Our results showed that, in late-pregnancy and early-lactation stages, hepcidin mRNA level was significantly reduced (Figure 5(a)). Combining with the observed higher expression of FPN1 in intestine, it suggests that the reduction in hepcidin level promoted the stability of FPN1 and then increased iron release from small intestine to blood as well as iron release from liver and spleen to the circulation. It seemed that the dramatically decreased iron stores, but not the plasmatic iron, mainly regulated the expression of hepcidin, as the serum iron concentration and transferrin saturation did not show any changes. It has been proposed that TfR2 is one of the iron sensors in liver that regulates hepcidin expression [25]. When Tf-Fe is at high level, it binds to TfR1 and releases HFE, which will then bind to TfR2 [26, 27]. Consequently, the HFE-TfR2 complex signals to active hepcidin transcription, possibly through HJV/BMP/SMAD signaling pathway [25]. Our results showed that liver TfR1 is increased (Figure 3(c)) and liver TfR2 is decreased (Figure 5(b)) during late-pregnancy and early-lactation stages, which may partially explain the reductions in hepcidin levels.

However, the regulatory mechanisms for hepcidin expression during pregnancy and lactation can be very complicated and involve multiple pathways. As we noticed, the changes of hepcidin expression started earlier than the reductions of liver iron content and liver TfR2. This may indicate that some other molecules expressed at early-pregnancy period can sense the onset of gestation and influence hepcidin expression. Estrogen has been found to affect hepcidin synthesis via a direct interaction with hepcidin mRNA [32], and testosterone also suppresses hepcidin transcription via unknown pathways [7]. The serum Cp level has been identified as an early indicator of pregnancy because of its raising expression at the very early time point of pregnancy [33, 34], and thereby it may also function in the regulation of hepcidin expression. In addition, the elevated erythropoiesis during pregnancy, particularly at late-pregnancy stage, might also be causative for the hepcidin suppression [25, 31]. The new discovered erythroferrone could be another important factor that directly suppresses hepcidin expression [35]. At the late stages of pregnancy, most of the pregnant mothers would experience different degrees of hypoxia; thus the transcription factor hypoxia-inducible factor (HIF) might also be a causative factor for hepcidin suppression [36, 37]. Besides the regulatory molecules in the maternal body, fetal iron status was also reported to regulate maternal iron metabolism during pregnancy in the rat [38]. Therefore, complicated mechanisms may exist for hepcidin regulation at each different stage of pregnancy and lactation, which needs to be investigated further in the future.

## 5. Conclusions

Our study comprehensively investigated the effects of pregnancy and lactation on the levels of iron stores, iron transport proteins, and iron metabolism regulatory molecules in rats. The potential regulatory role of hepcidin in iron metabolism in rat's pregnancy and lactation was explored, and its possible regulatory pathways were discussed. These results may contribute to the understanding of the abnormal iron metabolism during pregnancy and lactation at the molecular level and may provide experimental and theoretical bases for future studies to identify the key pregnancy-related regulators that cause these changes and to improve the iron-deficiency status for prepartum and postpartum women.

## Figures and Tables

**Table 1:** Serum iron status of rats at different stages of pregnancy and lactation.

	NP	9 dP	18 dP	1 dL	7 dL	14 dL	21 dL
SI (mg/L)	5.92 ± 0.77	7.25 ± 0.07	6.32 ± 1.24	6.35 ± 0.21	6.77 ± 1.01	7.24 ± 2.67	6.37 ± 0.94
TIBC (mg/L)	16.71 ± 1.39	17.57 ± 2.12	16.1 ± 4.06	15.1 ± 0.14	18.35 ± 1.45	16.32 ± 2.07	15.76 ± 1.03
UIBC (mg/L)	10.8 ± 1.65	10.8 ± 2.71	9.77 ± 4.58	8.75 ± 0.35	11.58 ± 1.17	9.07 ± 1.29	9.40 ± 0.41
TS (%)	0.33 ± 0.03	0.41 ± 0.06	0.42 ± 0.16	0.42 ± 0.02	0.37 ± 0.05	0.44 ± 0.12	0.40 ± 0.04
